# Positive Youth Development, Life Satisfaction, and Problem Behaviors of Adolescents in Intact and Non-Intact Families in Hong Kong

**DOI:** 10.3389/fped.2013.00018

**Published:** 2013-08-20

**Authors:** Daniel T. L. Shek, Hildie Leung

**Affiliations:** ^1^Department of Applied Social Sciences, The Hong Kong Polytechnic University, Hong Kong, China; ^2^Centre for Innovative Programmes for Adolescents and Families, The Hong Kong Polytechnic University, Hong Kong, China; ^3^Kiang Wu Nursing College of Macau, Macau, China; ^4^Department of Social Work, East China Normal University, Shanghai, China; ^5^Department of Pediatrics, Division of Adolescent Medicine, Kentucky Children’s Hospital, University of Kentucky College of Medicine, Lexington, KY, USA

**Keywords:** Chinese adolescents, intact families, non-intact families, positive youth development, life satisfaction, risk behavior

## Abstract

This study investigated whether Chinese adolescents living in intact and non-intact families differed in their positive development, life satisfaction, and risk behavior. A total of 3,328 Secondary 1 students responded to measures of positive youth development (such as resilience and psychosocial competencies), life satisfaction, and risk behavior (substance abuse, delinquency, Internet addiction, consumption of pornographic materials, self-harm, and behavioral intention to engage in problem behavior). Findings revealed that adolescents growing up in intact families reported higher levels of positive developmental outcomes and life satisfaction as compared with adolescents from non-intact families. Adolescents in non-intact families also reported higher levels of risk behaviors than those growing up in intact families.

## Introduction

The negative impacts of marital disruption on family processes and child developmental outcomes are outlined in different family theories. According to the structural family therapy perspective (1), family disruption would intensify enmeshment and psychological control of the parents. Family systems theories suggest that marital discords adversely impact the triadic relationship between the father, mother, and child in a family, whereby the collaborative effect of mother-father parenting (i.e., co-parenting) is greatly diminished (2). Similarly, family ecological theorists posit that marital disruption adversely impacts on adolescent development as a result of changing familial processes. Protective factors promote healthy adolescent development by buffering the adverse impacts of negative risk factors (3). Although adolescents spend increasing amount of time with their peers, one’s family continue to serve its adaptive function by providing a secure base for adolescents (4). A healthy family with both biological parents present yields better developmental outcomes for children based on the assumption that both parents are assets and serve their respective functions as sources of “emotional support, practical assistance, information, guidance, and supervision” [(5), p. 24]. In contrast, following marital disruption, family functions, and parental roles become problematic.

Social control theorists argue that adolescents from non-intact families exhibit problem behaviors because their bond to the society (including that with their family) becomes weak as an outcome of the changing family structure (6). It is believed that family restructuring, as a result of divorce or remarriage, causes weakening of parent-child attachment. Parents spend less time with their children and the level of direct parental supervision greatly diminishes, which makes it easier for adolescents to engage in delinquent behaviors. Hirschi (6) argued that parent-child relationships characterized by intimate communication where both parties are willing to share personal thoughts and feelings with each other create a psychological presence that serves as an impediment for children to committing delinquent acts. Barrett and Turner’s (7) study provides evidence for the social control theory in identifying stronger parental support, warmth and family cohesion, and more time spent with the family as significant protective factors against problem behaviors in adolescents. Unfortunately, parental monitoring is less effective as a protective factor within blended families, as facilitating factors, including rules, obligations, and boundaries, are often ambiguous (8). Hofferth and Anderson (9) also showed that children in single-parent families spent less time with their parents, and received less parental attention, supervision, and monitoring than those living with both biological parents.

The arguments of the social control theory are consistent with the social learning theory (10) which asserts that marital disruption would contribute to “lax parenting” which would further increase problem behavior in children and adolescents. Using the language of the attachment perspective, it is proposed that adolescents growing up in troubling family environments are less likely to be emotionally invested in the family itself. As a result, in order to seek for emotional attachment, they may become more involved in peer relationships instead. Deviant and unconventional models are more likely to occur within their peer groups (11).

Finally, stress theorists argue that the negative impact of growing up in non-intact family is mainly attributed to change. Parental divorce and remarriage result in changes in family structure which lead to modifications in family dynamics and roles causing immense stress to both the parents and the child. Pre- and post-divorce conflicts are commonly reported by children from broken homes. These conflicts directly impact on children through their influence on the emotional family environment they reside in. Adolescents who reported experiences of familial conflicts exhibited social withdrawal behavior and performed poorer on cognitive functions than did adolescents from intact families (12). In addition, adolescents in blended families also report stress arising from feelings of divided loyalty (e.g., liking a step-parent more than their biological parent) and discipline related issues (e.g., adjusting to new rules of the blended family) (13).

An integration of the existing theories on the impact of parental marital disruption on child developmental outcomes suggests that several mechanisms are involved. First, marital disruption adversely undermines the well-being of parents, which in turn subverts their roles as socialization agents. Second, marital disruption usually creates economic difficulty for the family. If the single-parent has to work as a result of financial difficulty, the quality of parental supervision is further diminished. If the family is on welfare, the negative impacts of economic disadvantage would also affect parenting. Third, as marital disruption leads to stressors in different domains, they would adversely influence parental efficiency and outcomes.

Empirically, research findings showed that compared to intact families, family processes are relatively poorer in non-intact families. Parents experiencing marital problems usually experience conflicts and display hostile behaviors which often cause negative emotions in their children (14, 15). Researchers have also found that parental marital disruption impaired parenting processes. Heath and MacKinnon (16) examined childrearing practices of divorced parents and found that the use of lax control among single mothers adversely impacted on the development of social competence in their children. Lone parents were not as demanding of their children and reported less restrictive rules in their parenting (17). In divorced, mother-custodial families, mothers became less effective monitors of the behaviors of their daughters as they matured (18). Furthermore, less expressive parent-child relationships, poor parental supervision, less positive parent-child interaction, and inconsistent parenting were also found in non-intact families (19, 20).

Parenting practices are also affected upon marriage dissolution. Following marital disruption, children are confused when parents maintained inconsistent rules or when one parent attempted to undermine the authority of the other parent (21). Shek (22, 23) also found that parental behavioral control processes were weaker and parent-child relational qualities were worse in non-intact than intact families. Children of parents who used less affective, unresponsive, disinterested, or negative parenting styles reported poorer psychological functioning (24).

There is empirical support for the thesis that parental marital disruption leads to poor adolescent developmental outcomes indexed by psychological symptoms. There are studies showing that children of non-intact families displayed more physical and mental problems than did children in intact families (25). With reference to different forms of families (including stable intact families, conflict intact families, single-parent families, and stepfamilies), adolescents from single-parent families performed worst in terms of different developmental outcomes, including physical health, suicidal ideation, mental health, relational well-being, and employment condition (26). As far as prevalence of psychological disorders is concerned, Halfon and Newacheck (27) reported that the prevalence of a poor mental health condition was lower in intact families than non-intact families.

Besides psychiatric morbidity, there are research findings showing that marital disruption was related to adolescent risk behavior. Research has generally found that adolescents from non-intact families were more likely to engage in deviant behaviors. For instance, in examining more than 2,000 parent-youth pairs in Australia, Mance and Yu (28) found that youths from non-intact families were more likely to smoke regularly and be in trouble with the police. Recently, Arkes (29) found that adolescents engaged in substance use at different stages of parental divorce, with a likely onset of alcohol use prior to the divorce and an increased risk of marijuana use right after the divorce. Alarming figures also revealed that students who scored low in family functioning measures (which commonly existed in non-intact families) were about twice as likely to report using alcohol as compared with their counterparts (30). Family disruptions not only increase the likelihood of adolescents’ externalizing behaviors, but are also associated with increasing levels of internalizing emotional and psychological problems, such as self-harm (31) and the presence of suicidal thoughts (32).

In addition to adolescent psychological morbidity and risk behavior, there are some isolated studies on the impact of marital disruption on positive youth development measures. The positive youth development approach is a strength-based approach to understand adolescent development which stresses the propensity for healthy development through the discovery and cultivation of strengths and potentials, such as resilience, self-esteem, and psychosocial competencies. There are studies showing that adolescents in intact families with lower levels of parental conflicts reported higher scores on religious and spiritual outcomes (33), resilience (34), behavioral, and emotional outcomes (16), as compared with those raised in non-intact families.

Life satisfaction is regarded as an integral part of adolescent well-being and an indicator of optimal functioning (35). As such, it would be important to look at life satisfaction of adolescents growing up in families with and without marital disruption. There is research showing that life satisfaction is negatively related to psychological symptoms (36) and risk behavior (37). Unfortunately, findings on the relationship between family structure and adolescent well-being remain inconclusive and sparse, although some researchers do suggest that children of single-parent families experience most ill-being (38), and changes in familial structure such as divorce have long-lasting adverse effects on adolescents’ well-being (22).

Against the above background, this study sought to gain insight on the impact of family structure on life satisfaction and developmental outcomes of adolescents in Hong Kong, since generalizations of findings from Western cultures to non-Western cultures should not be assumed without evidence. With the exception of Shek (39, 40), few studies have systematically examined the impact of family functioning on adolescent psychological well-being in Asia. Therefore, one of the goals of the present study is to contribute to the existing literature on family influences and perceived life satisfaction in adolescents by investigating their relation in an Asian context, particularly, with Chinese adolescents in Hong Kong. Given the fact that Hong Kong is a modern society (yet deeply rooted in traditional Chinese cultural values that place great emphasis on family), findings from the present research would shed light on the issue from the other side of the globe. Based on existing literature, it was hypothesized that adolescents from intact families would report better positive youth development outcomes, higher life satisfaction, and would be less likely to engage in problem behaviors, as compared to adolescents from disrupted families.

## Materials and Methods

### Participants and procedures

Project Positive Adolescent Training through Holistic Social Programs (P.A.T.H.S.) is a pioneering positive youth development program aimed at promoting holistic development among adolescents in Hong Kong. Participants in the current study were recruited from 28 participating schools randomly selected from all secondary schools in Hong Kong to join a longitudinal study. The sampling frame of the study was all Government and aided schools in Hong Kong. As the questionnaire was administered in Chinese, international schools and non-Chinese speaking schools were excluded from the present investigation. The present study reports findings from Wave 1 of the longitudinal data collection process. As such, only data from Secondary 1 students were included in the study. Among the 4,531 participants that were invited to complete the questionnaire, a total of 3,328 students participated, resulting in an overall response rate of 73.4%. With reference to the total Secondary 1 student population in Hong Kong at the time the study was conducted (67,963), the sample size can be considered as adequate (4.8% of the total population). Participants consisted of 1,719 males, 1,572 females, and 37 students did not indicate their gender. They were all Secondary 1 students, with a mean age of 12.59 years.

Data were collected at the schools, in a classroom setting, by trained research staff and/or school teachers with advance briefings. All participants responded en masse to all the instrument scales in the questionnaire in a self-administration format. Prior to data collection, ethical approval was obtained from The Hong Kong Polytechnic University, as well as parental and school consent. At the time of data collection, student consent were sought, and anonymity and confidentiality were emphasized during the administration process, and participants were given sufficient time to complete the questionnaire.

### Instruments

#### Assessment of household demographics

Marital Status of Parents. Respondents were asked to indicate the marital status of their parents on a four-alternative choice item; (a) divorced; (b) separated; (c) legally married husband and wife relationship; (d) other forms of marital relationship, such as cohabitation and re-married status. Respondents were asked to indicate the number of family members with whom they currently live with, including themselves but excluding their domestic helpers.

#### Assessment of positive youth development

Positive youth development was measured by the modified Chinese Positive Youth Development Scale (37). The scale has 44 items from 15 subscales tapping on the following constructs: bonding (three items; α = 0.74), resilience (three items; α = 0.79), social competence (three items; α = 0.86), recognition for positive behaviors (three items; α = 0.76), emotional competence (three items; α = 0.73), cognitive competence (three items; α = 0.81), behavioral competence (three items; α = 0.71), moral competence (three items; α = 0.73), self-determination (three items; α = 0.75), self-efficacy (two items; α = 0.65), clear and positive identity (three items; α = 0.78), beliefs in the future (three items; α = 0.84), prosocial involvement (three items; α = 0.80), prosocial norms (three items; α = 0.72), and spirituality (three items; α = 0.88). All subscales, with the exception of spirituality were measured using a 6-point scale, ranging from 1 (strongly disagree) to 6 (strongly agree). Spirituality was measured using a 7-point scale. A detailed description of the scale and its psychometric properties including validity and reliability analyses are reported in Shek and colleagues’ (41, 42) studies.

#### Assessment of life satisfaction

Life satisfaction of adolescents was assessed with Diener et al.’s (43) Satisfaction with Life Scale (LIFE) consisting of five items (α = 0.85). Respondents were asked to indicate on a 6-point scale, ranging from 1 (strongly disagree) to 6 (strongly agree), the extent to which they agreed that the statements described their feelings. A sample item includes, “If I could live my life over, I would change almost nothing.” A higher LIFE scale score indicates a higher level of life satisfaction.

#### Assessment of adolescent risk behavior

Problem behaviors were measured by seven measures. A final score was obtained by averaging the items in each measure. A higher score indicates a higher level of engagement (or intention to engage) in the problem behavior.

##### Substance use

Adolescents’ substance use was assessed using an eight-item 7-point scale (α = 0.50). Participants were asked to indicate their frequency, ranging from 0 (never) to 6 (daily), of using alcohol, tobacco, ketamine, cannabis, cough mixture, organic solvent, pills, and narcotics.

##### Delinquency

Adolescents’ delinquency was measured using a 12-item 7-point scale (α = 0.70). Respondents were asked to indicate their frequency of engaging in the stated behaviors in the past year, ranging from 0 (never) to 6 (more than 10 times). Antisocial behaviors included stealing, cheating, truancy, running away from home, vandalism, assault, engaging in sexual relationship with others, gang fighting, speaking foul language, staying away from home without parental consent, strong-arming others, and breaking into residences.

##### Intention of problem behavior engagement

Adolescents’ intention to engage in problem behaviors was measured with five items (α = 0.64). Respondents were asked to indicate on a 4-point scale, ranging from 1 (absolutely not) to 4 (absolutely will) whether they will engage in the problem behaviors, such as smoking, drinking, and gambling, in the next 2 years.

##### Internet addiction

Adolescents’ internet addiction behaviors were assessed with a 10-item two-choice, Yes-No, scale (α = 0.79). Participants were asked to indicate whether they experienced internet addiction behaviors, such as feeling depressed, anxious, or agitated without internet access, willingness to pay high internet service costs to stay online, spending exceedingly long hours on the internet, etc.

##### Consumption of sexual materials

Adolescents’ exposure to sexual materials was assessed using a 12-item 6-point scale (α = 0.91). Respondents were asked to report the frequency with which they have been exposed to the sexual materials in the past year, ranging from 0 (never) to 6 (daily). Sexual materials included those accessed via the internet or through traditional mass media such as magazines, books, and comics.

##### Deliberate self-harm

Adolescents’ behaviors of self-harm were assessed using the 17-item Deliberate Self-Harm Inventory [DSHI, (44)] (α = 0.83). Participants were asked to indicate on a two-choice, Yes-No, scale whether they have engaged in self-harm behaviors, such as cutting, burning, carving, bone-breaking, biting, and head-banging, in the past year.

##### Suicidal behaviors

Adolescents’ suicidal behaviors were measured using four items (α = 0.57). For instance, respondents were asked to self-report whether they have had suicidal ideations, and the number of times they have had such thoughts.

All scales were initially translated to Chinese by a professional bilingual translator and subsequently back translated to English by another experienced translator. Discrepancies between the English and Chinese versions were evaluated and reduced through an iterative review process by the translators and the first author.

### Data analyses

Preliminary analyses were conducted to examine whether there were differences in age, gender ratio, and the number of household members between the two groups (intact vs. non-intact families). Results revealed that the two groups differed in age and number of family members, but not in gender ratio. To assess the bias of the nested data (i.e., students were nested in schools), analyses using Linear Mixed Methods in SPSS were conducted. Results showed that the values of intra-class correlations for the outcome variables were lower than the cutoff of 0.25 suggested in the literature (45, 46). As such, it is legitimate to use generalized linear models.

Huberty and Morris (47) argued that the use of multivariate analyses of variance is appropriate in cases where the researcher has collected data for a system of conceptually interrelated dependent variables to address a multivariate hypothesis. As theoretical models and correlational analyses suggest significant intercorrelations among the dependent variables, multivariate analyses of covariance (MANCOVA) were conducted with family types (intact vs. non-intact families) as the independent variable and the positive youth development constructs, life satisfaction, and adolescents’ problem behaviors as dependent variables, treating age, and number of household members as covariates.

## Results

Intact families were operationalized as families in which parents of respondents are legally married husband and wife in their first marriage at the time of study (*n* = 2,781). Non-intact families were operationally defined as families in which parents were separated (*n* = 73), divorced (*n* = 209), re-married (*n* = 129), or engaged in other non-husband and wife relationships (*n* = 104). Table 1 provides a description of the data characteristics.

**Table 1:** **Descriptive statistics of respondents by family types**.

	Intact		Non-intact		Total	
Number of males	1437		263		1700	
Number females	1314		247		1561	

	***M***	**SD**	***M***	**SD**	***M***	**SD**

Age	12.57	0.80	12.73	0.73	12.59	0.74
Number of household members	4.14	1.01	3.53	1.31	4.04	1.09

Table 2 reports MANCOVA results. Several observations could be drawn from the analyses. First, with reference to positive youth development, adolescents from intact families reported significantly higher levels of positive youth development across 14 of the constructs as compared with their counterparts from non-intact families. Interestingly, however, adolescents from both intact and non-intact families did not differ in their recognition of positive behaviors. In terms of life satisfaction, adolescents from intact families were more satisfied with their lives than those from non-intact families. Third, youngsters coming from intact families were less likely to be engaged in problem behaviors as compared with those from non-intact families. Particularly, they reported lower levels of substance use, delinquency, intention to engage in problem behaviors, internet addiction, exposure to sexual materials, deliberate self-harm, and suicidal behaviors. The above findings are consistent with the predictions of the study and support thesis that family has impact on adolescent behaviors.

**Table 2:** **Effect of family types (intact vs. non-intact) on positive youth development, psychological well-being, and adolescent behaviors**.

	Intact families (mean scores)	Non-intact families (mean scores)	*F* value	Effect size
Positive youth development			Omnibus F 3.27**	0.018
Bonding	4.74	4.60	8.67*	0.003
Resilience	4.70	4.49	18.28**	0.007
Social competence	4.80	4.62	12.81**	0.005
Recognition for positive behavior	4.37	4.27	3.67	0.001
Emotional competence	4.31	4.09	17.78**	0.007
Cognitive competence	4.36	4.14	19.91**	0.007
Behavioral competence	4.57	4.41	13.80**	0.005
Moral competence	4.43	4.19	22.91**	0.009
Self-determination	4.51	4.34	13.10**	0.005
Self-efficacy	4.39	4.23	9.35*	0.004
Clear and positive identity	4.14	3.92	15.06**	0.006
Beliefs in the future	4.46	4.19	22.59**	0.008
Prosocial involvement	4.43	4.20	16.70**	0.006
Prosocial norms	4.69	4.53	10.06*	0.004
Spirituality	5.24	4.83	32.37**	0.012
**PSYCHOLOGICAL WELL-BEING**				
Life satisfaction	4.00	3.68	33.41**	0.011
Adolescent risk behaviors			Omnibus F 10.05**	0.033
Substance use	0.08	0.14	26.47**	0.010
Delinquency	0.36	0.50	29.69**	0.012
Problem behavioral intention	0.23	0.31	14.85**	0.006
Internet addiction	0.22	0.27	18.62**	0.007
Exposure to sexual materials	0.04	0.10	19.67**	0.008
Deliberate self-harm	0.03	0.06	22.90**	0.009
Suicidal behavior	0.05	0.10	31.14**	0.012

*p < 0.01, **p < 0.001.

## Discussion

The primary objective of the present study was to examine differences between adolescents in intact families and non-intact families in terms of different developmental outcome indicators. There are several unique features of this study. First, besides risk behavior, adolescent life satisfaction and positive youth development indicators were used. Hence, this can give us a comprehensive view of the problem area. Second, a large sample was employed. Finally, as there are few related studies in different Chinese contexts, Chinese adolescents were studied. Several observations are highlighted from the present study. First, consistent with our expectation, adolescents from intact families displayed higher levels of positive developmental outcomes across all positive youth development indicators than did adolescents from non-intact families, with the exception of a marginally significant difference yielded for adolescents’ recognition for positive behavior. This exception may be due to the fact that the items tap into whether adolescents perceive that their positive and prosocial behaviors are rewarded or complimented by their teachers or peers, which should be relatively less influenced by the adolescent’s immediate family structure. Second, in line with our expectation, adolescents from non-intact families reported a lower level of life satisfaction compared with their counterparts from intact families.

Third, adolescents from non-intact families were associated with higher levels of engagement in various risk behaviors, including substance use, delinquency, internet addiction, deliberate self-harm, and suicidal behaviors. In addition, they also reported relatively higher intention to be involved in risk behaviors, as well as more exposure to sexual materials. These findings are consistent with the predictions of the family ecological models, social control theories, and stress theory. These findings are also consistent with earlier studies demonstrating that adolescents who live with both their biological parents experience higher levels of life satisfaction compared to those from single-parent or restructured families (48). Similarly, the present findings are in line with those studies showing that adolescents residing in non-intact families are more likely to smoke, take drugs, or consume alcohol (49, 50).

Findings from the current study have both theoretical and practical implications. From a theoretical perspective, studies investigating the impact of family structure on adolescents’ development have been predominantly conducted in the West. Although family is integral in the lives of those in collectivistic cultures such as Hong Kong, in face of westernization and modernization, divorce rates have risen from 1.99 per 1,000 in 2000 to 2.57 in 2010 and the number of female single-parents rose markedly from 30,402 in 1996 to 57,613 in 2006 (51). Yet as Shek (52) astutely observed, there was a rarity of empirical findings on the impact of family structure on Chinese people. As such, the current findings broaden existing family literature by including an Asian sample. From a practical perspective, social workers, teachers, and parents should be aware of the impact of changing family structure on the development of adolescents and be sensitive to the potential developmental risks adolescents face in these turbulent environments.

Despite the unique contributions of the present study, readers should be cautious about the limitations of the study. Given that this study was conducted using cross-sectional data, a causal order is not to be assumed between family structure and positive youth development, psychological well-being, or problem behaviors among adolescents. In fact, it is important to collect longitudinal data if we are interested in looking at the impact of the changing family structure on adolescent development. Furthermore, it should be noted that the current study did not account for possible differences in the quality of family relationships or parenting of intact and non-intact families. Thus, future studies should be conducted to investigate the mediating roles of parent-child relational qualities, perceived parental control processes, or parenting styles on the family structure and adolescent development relation. Although the patterns of data yielded in this study are consistent with those in the West demonstrating the importance of a healthy family to adolescent development and well-being, the sample of this study was confined to Hong Kong adolescents. As such, further replications in other Chinese communities are needed before generalizations can be made to the entire Asian population. Lastly, as adolescent developmental outcomes were assessed via self-report measures, the inclusion of more diverse sources of information (e.g., from parents, teachers) would allow for external validation and better understanding of the issue.

The present study demonstrates clearly that adolescents in non-intact families experience lower levels of psychological well-being, poorer positive youth development, and are more likely to engage in problem behaviors, as compared with those from healthy intact families. In view of the scanty scientific literature in the Chinese contexts, this study contributes to the field by illustrating the impact of family structure on adolescent development in an Asian context. Future studies should examine the intricate familial and psychological processes underlying this observation.

## Conflict of Interest Statement

The authors declare that the research was conducted in the absence of any commercial or financial relationships that could be construed as a potential conflict of interest.
